# Angiotensin II Receptor 1 Blockage Limits Brain Damage and Improves Functional Outcome After Brain Injury in Aged Animals Despite Age-Dependent Reduction in AT1 Expression

**DOI:** 10.3389/fnagi.2019.00063

**Published:** 2019-04-26

**Authors:** Ralph Timaru-Kast, Philipp Gotthardt, Clara Luh, Changsheng Huang, Regina Hummel, Michael K. E. Schäfer, Serge C. Thal

**Affiliations:** ^1^Department of Anesthesiology, University Medical Center of the Johannes Gutenberg University, Mainz, Germany; ^2^Center for Molecular Surgical Research, University Medical Center of the Johannes Gutenberg University, Mainz, Germany; ^3^Focus Program Translational Neurosciences, University Medical Center of the Johannes Gutenberg University, Mainz, Germany

**Keywords:** angiotensin II receptor type 1, traumatic brain injury, aged, inflammation, candesartan, AT1 inhibition, neutrophil granulocyte, M2 microglia polarization

## Abstract

Traumatic brain injury (TBI) is a frequent pathology associated with poor neurological outcome in the aged population. We recently observed accelerated cerebral inflammation in aged mice in response to TBI. Candesartan is a potent specific inhibitor of angiotensin II receptor type 1 (AT1) which limits cerebral inflammation and brain damage in juvenile animals after experimental TBI. In the present study, we show significantly lower posttraumatic AT1 mRNA levels in aged (21 months) compared to young (2 months) mice. Despite low cerebral *At1* expression, pharmacologic blockade by treatment with candesartan [daily, beginning 30 min after experimental TBI by controlled cortical impact (CCI)] was highly effective in both young and aged animals and reduced histological brain damage by −20% after 5 days. In young mice, neurological improvement was enhanced by AT1 inhibition 5 days after CCI. In older animals, candesartan treatment reduced functional impairment already on day 3 after TBI and post-traumatic body weight (BW) loss was attenuated. Candesartan reduced microglia activation (−40%) in young and aged animals, and neutrophil infiltration (−40% to 50%) in aged mice, whereas T-cell infiltration was not changed in either age group. In young animals, markers of anti-inflammatory microglia M2a polarization [arginase 1 (*Arg1*), chitinase3-like 3 (*Ym1*)] were increased by candesartan at days 1 and 5 after insult. In older mice 5 days after insult, expression of *Arg1* was significantly higher independently of the treatment, whereas *Ym1* gene expression was further enhanced by AT1 inhibition. Despite age-dependent posttraumatic differences in *At1* expression levels, inhibition of AT1 was highly effective in a posttreatment paradigm. Targeting inflammation with candesartan is, therefore, a promising therapeutic strategy to limit secondary brain damage independent of the age.

## Introduction

Traumatic brain injury (TBI) is the most common cause for trauma-related death and severe disability in industrialized countries (Langlois et al., 2006). Incidence of TBI peaks between 15 and 24 and after 75 years of age (Flaada et al., 2007). Aging patients show remodeling of the immune system that encompasses changes in composition, phenotype and function of immune cells, as well as the development of a subclinical chronic and systemic pro-inflammatory state. As a result, advanced age is associated with poor outcome following TBI (Stocchetti et al., 2012) and is characterized by elevated brain tissue and blood serum inflammatory cytokines levels (Hazeldine et al., 2015) and enhanced immune response (Csiszar et al., 2003). Aging rodents show an early and long-lasting posttraumatic cytokine release (Sandhir et al., 2004), pronounced and prolonged microglia activation (Sandhir et al., 2008), and immune cell infiltration (Neumann, 2001). Accordingly, we recently demonstrated an enhanced cerebral inflammatory response, brain edema formation and functional deficits after experimental TBI in 21- compared to 2-months-old mice (Timaru-Kast et al., 2012a). The data suggest that early onset and long duration of inflammation may be important for poor functional recovery in older animals.

One of the most important mediators of cerebral inflammation is angiotensin II (AII; Villar-Cheda et al., 2014), the main effector of the intrinsic cerebral renin-angiotensin system (RAS). AII mediates vasoconstriction and blood pressure regulation by activation of the AII receptor type 1 (AT1). Besides influence on vascular tone, AT1 activation is involved in different pathophysiologic processes, including neurodegenerative diseases, neuronal injury, and cognitive disorders (Saavedra et al., 2004; Villapol et al., 2012; Villar-Cheda et al., 2014; Trigiani et al., 2018). After cerebral insults, AT1 mediates inflammation and vasoconstriction, thereby aggravating secondary brain damage (Culman et al., 2002; Timaru-Kast et al., 2012b). AT1 signaling is mediated by mitogen-activated protein kinases (MAPKs; ERK1/2, JNK, and p38MAPK), glycogen synthase kinase, Rho/ROCK kinase, receptor tyrosine- (PDGF and EGFR) and non-receptor tyrosine-kinases (Src, Pyk2, and JAK/STAT; Borrajo et al., 2014b). AT1 mediated activation of nicotinamide adenine dinucleotide phosphate (NADPH)-dependent oxidases is a major source of oxidative stress, enhances neuroinflammation and is relevant in the pathogenesis of age-associated degenerative diseases (Rey et al., 2007). In animal models of aged, increased AT1 expression and activation was associated with elevated levels of inflammatory cytokines, e.g., interleukin 1β (IL-1β) and tumor necrosis factor α (TNFα; Villar-Cheda et al., 2012). In contrast, the AII receptor type 2 (AT2) triggers vasodilatation, anti-inflammation and neuroregeneration that can alleviate pathological processes (Joseph et al., 2014; Umschweif et al., 2014).

Brain RAS is involved in neuroinflammatory processes associated with age (Labandeira-Garcia et al., 2017). Therefore, in the present study, we tested the hypothesis that inhibition of AT1 is an effective neuroprotective strategy against age-related exacerbated cerebral inflammation after TBI.

## Materials and Methods

### Animals

Young male C57Bl6N (2 months; 21 ± 1 g) and aged (21 months; 40 ± 5 g) mice (Charles River Laboratory, Sulzfeld, Germany) were randomly assigned to experimental groups^1^. Experiments and analyses were performed by investigators blind towards treatment. The Animal Ethical Care Committee of the Regional Authority in Rhineland-Palatinate approved all experiments (protocol number: G08-1-012). The experiments were performed in compliance with institutional and national guidelines. Animals were kept under controlled light and environmental conditions (12 h dark/light cycle, 23 ± 1°C, 55 ± 5% relative humidity) and free access to food (Altromin, Germany) and water.

#### Experimental TBI and Anesthesia

Animals were anesthetized by intraperitoneal application of midazolam (Ratiopharm, Germany), fentanyl (CuraMed, Germany) and medetomidine (Pfizer, Germany). An air mixture (40% O_2_ and 60% N_2_) was supplied *via* facemask in spontaneously breathing mice (Thal and Plesnila, 2007). The depth of anesthesia was verified by respiration rate and pedal withdrawal reflexes. Rectal temperature was maintained constant at 37°C by a feedback-controlled heating pad (Hugo Sachs, Germany). TBI was performed by controlled cortical impact (CCI) as previously described in detail (Timaru-Kast et al., 2012b). Briefly, the animal’s head was fixed in a stereotactic frame (Kopf Instruments, Tujunga, LA, USA) and a large craniotomy (4 mm × 4 mm) was drilled above the right parietal cortex between the sagittal, lambdoid, and coronal sutures and the insertion of the temporal muscle. A custom-fabricated controlled pneumatic impactor (L. Kopacz, Mainz, Germany) was placed perpendicularly to the brain surface and the impactor tip (diameter, 3 mm) centered in the middle of the craniotomy. The impact parameters were as follows: velocity, 8 m/s; duration, 150 ms; brain penetration, 1 mm. Immediately after CCI, the craniotomy was closed with conventional tissue glue (Histoacryl; Braun-Melsungen, Melsungen, Germany) and filament sutures. After the procedure animals were placed in their individual cages and allowed to recover for 6 h in an incubator heated to 33°C at a humidity of 35% (IC8000, Draeger, Germany).

### Application of Candesartan or Vehicle Solution

The crystalline form of the active drug candesartan (CV-11974, Takeda Pharma, Japan) was dissolved prior to each set of experiments in 0.037 M Na_2_CO_3_ (vehicle solution) in a concentration of 10 μg/ml. The animals received 0.1 mg/kg candesartan or vehicle solution by subcutaneous injection 30 min after insult, followed by a daily injection for four consecutive days after insult.

### Experimental Protocols

#### Regulation of RAS Marker Genes Following TBI

*At1a*, *At1b* and *At2* mRNA expression were determined by quantitative real-time PCR (qPCR) in naïve animals (young and old: *n* = 6 each), 24 (young and old: *n* = 7 each) and 72 h after CCI (young: *n* = 7; old: *n* = 9; seven survived).

#### Influence of Age on AT1 Mediated Protection After TBI

Mice subjected to CCI were randomly assigned to vehicle or candesartan treatment [young: vehicle and candesartan (*n* = 8 each); old: vehicle (*n* = 10; seven survived) and candesartan (*n* = 8; seven survived), young and old naïve (*n* = 6 each)]. Treatment started 30 min after TBI and was repeated daily until postoperative day 4. Lesion volume, expression of microglia activity markers, cytokines and *At1a* expression levels, the number of activated microglia, lymphocyte and neutrophil infiltration were determined at 5 days after insult.

#### Influence of Age on AT1 Mediated Brain Edema Formation and Expression of Cytokine and Microglial Markers at 1 Day After TBI

Brain water content was determined 24 h post-trauma in young and old animals treated with candesartan (young *n* = 8; old *n* = 7) or vehicle (young *n* = 8; old *n* = 7). In addition, perilesional cytokine and microglia marker expression was quantified by qPCR.

### Physiological Parameters

Blood pressure was measured 5 min before and after CCI at the tail using a modified NIBP system (RTBP 2,000, Kent, USA) as previously described in detail (Thal and Plesnila, 2007). Additionally, blood pressure values were determined daily for 8 days before (training phase) and for 4 days after CCI and candesartan or vehicle treatment. Perioperative body temperature was measured by a rectal temperature probe (Physitemp; Clifton, NJ, USA).

### Assessment of Functional Outcome

The neurological outcome was determined by modified neurological severity score (mNSS; modified after Tsenter et al., 2008) 1 day before and 24, 72, and 120 h after CCI by an investigator blind toward the group allocation. To calculate mNSS, general behavior, alertness, motor ability and balance were rated with 6 different tasks (Tsenter et al., 2008). Each task was scored from 0 (normal) up to 3 (failed task). The mNSS ranges from 0 (healthy) to 15 (severely impaired) points (Tsenter et al., 2008; Thal et al., 2013; Table 1).

**Table 1:** Modified Neurological Severity Score (mNSS).

		Points
**1. Exit from circle?**	<30 s	0
	For 30 s	1
	For 60 s	2
	>2 min	3
**2. Startle reflex**	Present	0
	Absent	1
**3. General behavioral deficit?**		
Seeking behavior	Present	0
	Absent	1
Walk straight	Present	0
	Absent	1
**4. Coordination** (Criteria: 0P: no impairment; 1P: feet misplacement, unstable; 2P: stops moving)		
Beam walking 3 cm	Score	(0–2)
Beam walking 1.5 cm	Score	(0–2)
Beam walking 1 cm	Score	(0–2)
**5. Balance** (Criteria: 0P: grips stick with four paws; 1P: 1–4 paws not gripping)		
Round stick	Score	(0–1)
Square stick	Score	(0–1)
**6. Motor deficit**		
Hemiparesis	Present	0
	Absent	1

*The modified Neurological Severity Score was designed on the basis of the Neurological Severity Score introduced by Tsenter et al. (2008). The mNSS focusses on motoric function and behavioral deficits and was performed 1 day before CCI and on posttraumatic day 1, 3 and 5 after experimental traumatic brain injury (TBI)*.

### Histologic Evaluation of Brain Damage and Immunohistochemistry

At the end of the observation period, brains were removed in deep anesthesia. For tissue evaluation, the brains were frozen in powdered dry ice after dissection and stored at −20°C. They were then cut in the coronal plane with a cryostat (HM 560 Cryo-Star; Thermo Fisher Scientific, Germany) as previously described in detail (Timaru-Kast et al., 2012b). The first slide was defined according to the first section corresponding to bregma +3.14 mm in the Mouse Brain Library^2^. Sixteen Sections (10 μm) were collected at 500 μm-intervals and placed on Superfrost+TM slides (Thermo Fisher Scientific). In cresyl violet stained sections, the total area of both hemispheres and the injured brain tissue area were determined for each section and animal using a computerized image analysis system (Delta Pix Insight; Maalov, Denmark) by an investigator blind to the group allocation. The total hemispheric brain volumes and the lesion volumes were calculated by following formula: 0.5 (mm) × [area of slide 1 (mm^2^) + area of slide 2 (mm^2^) + … + area of slide 16 (mm^2^)]; Timaru-Kast et al., 2012b. Immunohistochemistry was performed as described before (Huang et al., 2016). For immunohistochemical staining, cryosections were fixed in 4% paraformaldehyde in phosphate buffered saline, incubated with blocking solution containing serum (5% goat serum, 2% bovine serum albumin, Gibco) and 0.1% TX-100 (Sigma) in PBS for 1 h at room temperature (RT). Primary antibodies specific for ionized calcium-binding adapter molecule-1 (Iba-1, rabbit anti-mouse, anti-Iba-1 antibody; Wako Chemicals, Neuss, Germany), CD3 (rat anti-CD3, Zytomed, Germany) or Gr1 (mouse anti-Ly6G, Abcam, UK) were applied in blocking solution overnight at 4°C. The sections were washed, incubated with secondary biotin-conjugated antibodies (goat anti-rabbit IgG; Merck; Darmstadt, Germany) and processed according to the manufacturer’s instructions using a Vectastain Elite ABC Kit (Vector Laboratories, Burlingame, CA, USA). Images were taken at 20× magnification (Axiovert, Zeiss, Germany). The total number of positive cells (+) was counted at bregma −1.28 mm by two investigators blinded to randomization using ImageJ software (National Institutes of Health, USA). Iba1- and CD3-positive cells were assessed in a region of interest (ROI) of 0.52 mm × 0.65 mm in the cortical tissue adjacent to the lesion. Activated microglia were identified as Iba-1-immunolabeled cells with bushy-round shaped morphology with phagocytic, amoeboid appearance (Donat et al., 2017). Because neutrophil infiltration was predominantly present in the contused tissue with very few positive cells in the perilesional area, Gr1-positive cells were counted within the lesion. Therefore, using ImageJ, the number of cells in each section was calibrated to the measured lesion area resulting in a relative number (n/mm^2^).

### Quantification of Brain Water Content

After removal of the brains 24 h after CCI, hemispheres were cut along the interhemispheric plane. For gene expression analysis thin coronal slices of both hemispheres were taken. Thereafter both hemispheres were weighed to assess their wet weight (WW) and then dried by speed vacuum as described in detail (Sebastiani et al., 2017) to determine the dry weight (DW). Tissue water content was obtained by (WW − DW)/WW × 100 [%].

### Gene Expression Analysis With Quantitative Real-Time PCR (qPCR)

At the end of the observation period, animals were euthanized in deep anesthesia by cervical dislocation, and brains were carefully removed and gently placed in dry ice. After tissue sampling, RNA extraction and cDNA synthesis qPCR were performed. All assays were carried out in our laboratory by an investigator blinded to group allocation. Using mouse-specific primers and probes and optimized temperature conditions for qPCR (Timaru-Kast et al., 2012a,b, 2015; Tables s 2A,B), absolute copy numbers of the target genes *At1a*, *At1b*, *At2*, arginase 1 (*Arg1*), chitinase3-like 3 (*Ym1*), *Tnfa* (for TNFα), *Il1b* (for IL-1β) interleukin 6 (*Il6* for IL-6), inducible (*iNos*) and endothelial nitric oxide synthase (*eNos*) were calculated, and were then normalized against the absolute copy numbers of the reference gene cyclophilin A (*Ppia*; Timaru-Kast et al., 2015).

**Table 2A:** Primers and probes for quantitative real-time PCR.

PCR assay primer (annealing temperature)	Oligonucleotide sequence (5′-3′)	Amplicon length (Bp)	GenBank No.
***Ppia* (55°C)**			
*Ppia* F	GCGTCTSCTTCGAGCTGTT	146	NM_008907
*Ppia* R	RAAGTCACCACCCTGGCA		
*Ppia* FL	GCTCTGAGCACTGGRGAGAAAGGA-FL		
*Ppia* Cy5	Cy5-TTGGCTATAAGGGTTCCTCCTTTCACAG-PH		
***Il1b* (55°C)**			
*mIl1b* S	GTGCTGTCGGACCCATATGAG	348	NM_008361
*mIl1b* A	CAGGAAGACAGGCTTGTGCTC		
*mIl1b* FL	TAATGAAAGACGGCACACCCACCC		
*mIl1b* LC 610	Red610-CAGCTGGAGAGTGTGGATCCCAAGC-PH		
***Il6* (58°C)**			
*mIl6* F(ex2,3)	TCGTGGAAATGAGAAAAGAGTTG	471	NM_031168
*mIl6* R(ex5,6)	TATGCTTAGGCATAACGCACTAG		
*mIl6* FL	CATAAAATAGTCCTTCCTACCCCAATTTCC-FL		
*mIl6* Cy5	Cy5–TGCTCTCCTAACAGATAAGCTGGAGTCAC-PH		
***Tnfa* (62°C)**			
*mTnfa* F	TCTCATCAGTTCTATGGCCC	212	NM_013693
*mTnfa* R	GGGAGTAGACAAGGTACAAC		
***iNos* (58°C)**			
*Nos2* F	TGTGTCAGCCCTCAGAGTAC	312	NM_010927
*Nos2* R	CACTGACACTYCGCACAA		
*Nos2* FL	GAAGCCCCGCTACTACTCCATC-FL		
*Nos2* LC 640	Red640-GCTCCTCCCAGGACCACACCC-PH		

*Mouse specific primers and probes and optimized temperature conditions for quantitative real-time polymerase chain reaction (qPCR): cyclophilin A (*Ppia*), interleukin 1β (*Il1b*), interleukin 6 (*Il6*), tumor necrosis factor α (*Tnfa*), inducible nitric oxide synthase (*iNos*); F: forward, R: reverse. Kits (manufacturer): *Ppia*: Absolute Fast qPCR Mix AB-4325 (Abgene); *Agtr1* (*At1a*), *Agtr1b* (*At1b*), *Agtr2* (*At2*): Maxima Probe qPCR Master Mix K0262 (Fermentas); *Il1b*, *Il6*, *eNos*, *iNo*s: Light Cycler 480 Probes Master 04887301001 (Roche); *Tnfa*, *Arg1* and *Ym1*: Absolute Blue qPCR SybrgreenMix AB-4166 (Thermo Scientific)*.

**Table 2B:** Primers and probes for quantitative real-time PCR.

PCR assay primer (annealing temperature)	Oligonucleotide sequence (5′→3′)	Amplicon length (Bp)	GenBank No.
***At1a* (55°C)**			
*Agtr1* S	GCAAAGCTGTCTTACATTAATAGAT	121	NM_177322
*Agtr1* A	AATCAAAAGGAGACCGCT		
*Agtr1* FL	CCACAAATCCATCCAgCTCCT-FL		
*Agtr1* LC 640	Red640-ACTTGTCCTTGGGGCAGCCA-Ph		
***At1b* (55°C)**			
*Agtr1b* F	GTGTGTTAAGATTTGCTAGGC	208	NM_175086
*Agtr1b* A	TgAAATAAAACTGGTCCAACTAT		
*Agtr1b* FL	CATGCATTACCTCAGTCATAAAGTCAA–FL		
*Agtr1b* LC 640	Red640-CTGCTGTGATTCTCTCCCAGGT–PH		
***At2* (55°C)**			
*Agtr2* S	GGGGAGTAGTTTGAATCTGC	154	NM_007429
*Agtr2* A	GGTAACACAGCTTCAGGTATTATATT		
*Agtr2* FL	TCGTGTTAAGAATGAGTTCTGTGGACC-FL		
*Agtr2* LC 640	Red640-GGGCTTGCTTTAAATCACCTTCACAG-PH		
***eNos* (55°C)**			
*eNos* F	TCCGATTCAACAGTGTCTCC	306	NM_008713
*eNos* R	CCACACAGAAGGTTTCACAG		
*eNos* FL	TCCGATT CAACA GTGTCT CCTGC-FL		
*eNos* Cy5	Cy5-TCAGACCCACTGGTATCCTCTTGG-PH		
***Arg1* (58°C)**			
*Arg1* F	CTCCAAGCCAAAGTCCTTAGAG	185	NM_007482
*Arg1* R	AGGAGCTGTCATTAGGGACATC		
***Ym1* (58°C)**			
*Ym1* F	CAGGTCTGGCAATTCTTCTGAA	197	M94584
*Ym1* R	GTCTTGCTCATGTGTGTAAGTGA		

*Mouse specific primers and probes and optimized temperature conditions for quantitative real-time polymerase chain reaction (qPCR): angiotensin II receptors type 1A (*At1a*), 1B (*At1b*) and 2 (*At2*), arginase 1 (*Arg1*), chitinase-like protein 3 (*Ym1*), endothelial nitric oxide synthase (*eNos*); F: forward, R: reverse. Kits (manufacturer): *Ppia*: Absolute Fast qPCR Mix AB-4325 (Abgene); *Agtr1* (*At1a*), *Agtr1b* (*At1b*), *Agtr2* (*At2*): Maxima Probe qPCR Master Mix K0262 (Fermentas); *Il1b*, *Il6*, *eNos*, *iNos*: Light Cycler 480 Probes Master 04887301001 (Roche); *Tnfa*, *Arg1* and *Ym1*: Absolute Blue Qpcr Sybrgreenmix AB-4166 (Thermo Scientific)*.

#### Tissue Sampling, RNA Isolation and cDNA Synthesis

Brain tissue samples were excised during histological cryosectioning (−22°C) from bregma 0.7 mm to bregma −3.7 mm (area of injury) as previously described (Luh et al., 2010). Samples from lesion and perilesional tissue (right upper quadrant) and corresponding contralateral cortical tissue (left upper quadrant) were separately collected, snap frozen in liquid nitrogen, and stored at −80°C (Luh et al., 2010). RNA extraction, cDNA synthesis and quantitative real-time PCR were performed with standard protocols as previously described in detail (Thal et al., 2008; Luh et al., 2010). Samples were lysed with Qiazol-reagent (Qiagen, Hilden, Germany) and homogenized with a MM300 mill mixer (Retsch, Haan, Germany) operated at 30 Hz for 2 min. Total RNA was isolated using RNeasy Lipid Tissue Mini Kit (Qiagen) according to the manufacturer’s instructions and eluted with 30 μL of RNase-free water. The RNA concentration was determined spectrometrically with the NanoVue system (GE Healthcare Europe, Munich, Germany). Thereafter, 0.5 μg extracted RNA was reverse-transcribed into cDNA by the Verso cDNA Kit (ABgene, Hamburg, Germany) according to the manufacturer’s instructions. cDNA of each sample was amplified by a real-time Lightcycler 480 PCR System (Roche).

#### PCR Standard Generation for Absolute qPCR Quantification

PCR fragments of all applied genes were generated by PCR on an Eppendorf Thermocycler gradient (Eppendorf, Hamburg, Germany). PCR cycling parameters were as follows: thermal activation for 10 min at 95°C and 50 cycles of PCR (melting for 45 s at 94°C, annealing for 45 s at 55–65°C, and extension for 60 s at 72°C). Applied primers are listed in Tables s 2A,B. To verify the specificity of the PCR reaction, PCR products were electrophoresed alongside the 50 bp DNA Molecular Weight Marker XIII (Roche Diagnostics, Mannheim, Germany) through a 2% (w/v) agarose gel (Invitrogen). The gels were stained with SYBR green (Roche), and images were captured using a Kodak EDAS 120 Image System (Eastman Kodak Sàrl, Genève, Switzerland). The PCR products were purified with QIA quick PCR Purification Kit (Qiagen) according to the manufacturer’s instructions, and the DNA concentration was determined using NanoVue. The copy number was calculated and serial 10-fold dilutions were made in the range of 1 × 10^7^–1 × 10^1^ copies.

#### Efficiency and Linearity of Real-Time PCR Amplification

A standard curve for absolute quantification was generated with PCR DNA for each PCR product (10^1^–10^7^), showing similar and good efficiency (90–110%) and linearity. Real-time PCR efficiencies were calculated from the given slopes with LightCycler software. The number of crossing point cycles (Cp) vs. logarithmic DNA concentration (reverse transcribed total RNA) in the range of 1 × 10^1^ to 1 × 10^7^ DNA copies/μL were plotted to calculate the slope (S). The corresponding real-time PCR amplification factor efficiencies (E) were calculated according to the equation: *E* = 10^−1/slope^. Ideally, the efficiency of a PCR should be 100%, meaning that for each cycle, the amount of product doubles (amplification factor, *E* = 2; Pfaffl, 2001). Calibration curves (software version: LCS480 1.5.0.39), real-time PCR slope and efficiency values were within the desired range (90–110%). Values were as follows: *Ppia*: 39.81, 1.923, −3.523; *Il1b*: 36.39, 1.950, −3.448; *Il6*: 37.69, 1.907, −3.568; *Tnfa*: 35.27, 1.919, −3.532; *iNos*: 38.56, 1.917, −3.539; *At1a*: 36.80, 1.920, −3529; *At1b*: 37.17, 1.895, −3.602; *At2*: 38.92, 1.948, −3.453; *eNos*: 42.59, 1.714, −4.272; *Arg1*: 41.29, 1.898, −3.592; *Ym1*: 41.22, 1.870, −3.677 for γ intercept:, efficiency and slope, for all target genes, respectively. The standard curves showed high linearity (Pearson correlation coefficient *r* > 0.95).

#### The Applied Kits (Manufacturers)

*Ppia*: Absolute Fast qPCR Mix AB-4325 (Abgene); *At1a*, *At1b*, *At2*: Maxima Probe qPCR Master Mix K0262 (Fermentas); *Il-1b*, *Il-6*, *eNos*, *iNos*: Light Cycler 480 Probes Master 04887301001 (Roche); *Tnfa*, *Arg1* and *Ym1*: Absolute Blue qPCR SybrgreenMix AB-4166 (Thermo Scientific).

#### Quantitative (Real Time) PCR (qPCR)

Equal amounts of cDNA (1 μL) of each sample were analyzed in duplicates and amplified by real-time Lightcycler 480 PCR System (Roche). Real-time RT PCR kits were used according to the manufacturer’s instructions. Real-time cycling parameters were as follows: thermal activation for 10 min at 95°C and 50 cycles of PCR (melting for 10 s at 95°C, annealing for 10 s at 55°C (for HybProbe assays) or according to Tables s 2A,B, extension for 15 s at 72°C). Applied primers and probes are listed in Tables s 2A,B. The absolute copy numbers of the target genes were normalized against the absolute copy numbers of cyclophilin A (*Ppia*) as the reference gene.

#### Choice of Reference Gene

The reference gene *Ppia* was shown to be stable in an experimental setting (Bustin, 2002; Huggett et al., 2005) and chosen as single normalizer for both age groups based on our recent findings in young compared to aged mice after experimental TBI (Timaru-Kast et al., 2015). In order to improve comparability of the mRNA expression data between studies and groups and to eliminate qPCR kit dependent differences and limitations, qPCR data was normalized with PPIA and then related to normalized naïve target gene expression from naïve tissue samples from the corresponding brain region (Garcia-Bardon and Thal, 2016). Therefore, normalized target gene expression values are expressed as % naïve expression.

### Statistical Analysis

All experiments were randomized and performed by investigators blinded towards treatment groups (computer-based randomization). To determine the required sample size, an* a priori* power analysis using G*Power was performed using lesion volume data from previously published studies (Faul et al., 2009). The* a priori* power analysis was performed to determine an effect size of 0.7, standard statistical power (1-β) of pβ = 0.95 and a significance level (α) of 0.05. Statistical analysis was performed using Sigma Plot 14 (Systat Software, Germany) and GraphPad Prism 8 Statistical Software (GraphPad Software Inc., La Jolla, CA, USA).

In the first investigation with the assessment of RAS marker gene regulation following TBI in untreated young and old mice, mRNA expression data were compared between experimental groups with Mann Whitney Rank Sum Tests, *p* values adjusted for multiple comparisons (Bonferroni-Holm correction). In experimental groups where age and treatment factors are present, a two-way analysis of variance (two-way ANOVA) was performed: physiologic data, lesion volumes, brain water content, number of activated microglia, Gr1^+^ as well as CD3^+^-cells and mRNA expression data were compared between experimental groups with two-way ANOVA and *post hoc* with all-pairwise multiple comparison procedures (Holm-Sidak method). Because 3/10 vehicle-treated and 1/8 candesartan-treated aged mice died before the end the experimental observation period, there were missing values in the repeated measures dataset and a two-way repeated measures ANOVA (two-factor repetition) was not possible. Therefore, repeated measures of body weight (BW), blood pressure and mNSS data were analyzed with the mixed effect model using the restricted maximum likelihood method with Holm Sidak’s multiple comparison test.

The *p* values were adjusted for multiple comparisons. Values of *p* < 0.05 were considered significant. Data are presented as mean and standard deviation (mean ± SD).

## Results

### Perioperative Physiological Parameters Were Unchanged Between Young and Old Mice

Peri- and intraoperative body temperature (rectal measurement) was within physiologic range and there was no difference between groups (Table 3). Intraoperative systolic blood pressure values (after CCI induction under general anesthesia) were within physiological limits (Table 4). The values during surgery were higher in young compared with old mice.

**Table 3:** Intraoperative body temperature and pre- and postoperative body weight.

	Temperature	g BW	g BW	g BW	g BW	g BW	g BW
	[°C]	Before	1 dpi	2 dpi	3 dpi	4 dpi	5 dpi
Age 2 months Vehicle	*36.6 ± 0.4*	20.4 ± 0.8*	19.6 ± 1.3*	20.2 ± 1.9*	21.1 ± 1.0*	21.4 ± 0.6*^#^	22.0 ± 0.7*^##^
Age 2 months Candesartan	*36.7 ± 0.4*	20.7 ± 0.5*	19.1 ± 1.0*^#^	20.0 ± 1.1*	20.7 ± 1.1*	21.1 ± 0.5*	21.5 ± 0.8*
Age 21 months Vehicle	*36.5 ± 0.4*	40.2 ± 5.6*	39.1 ± 5.3*^#^	37.5 ± 5.3*^##^	36.7 ± 4.8*^##^	37.2 ± 4.9*^#^	36.8 ± 4.6*^#^
Age 21 months Candesartan	*36.5 ± 0.5*	39.5 ± 3.4*	38.7 ± 2.6*	37.3 ± 2.7*	36.6 ± 2.5*	36.3 ± 2.1*	36.5 ± 1.5*

Perioperative rectal temperature under general anesthesia (in italic characters) was at physiologic levels. Body weight (BW) before and during 5 days after experimental brain trauma (days post-injury, dpi) in grams (g) was different between age groups (*p < 0.001 old vs. young), but not affected by treatment in young mice. However, in aged mice, candesartan led to a stabilization of bodyweight with less marked decrease after 1 dpi, whereas in vehicle-treated old mice body weight decreased constantly (^#^*p* < 0.05 and ^##^*p* < 0.001 vs. before CCI; two-way ANOVA with repeated measures and mixed effect analysis, Holm Sidak’s multiple comparisons test; data is presented as mean ± SD).

**Table 4:** Systolic blood pressure after injury.

Hours after CCI	0 (CCI)	6 h	24 h	48 h	72 h	96 h	120 h
Age 2 months Vehicle	133 ± 14*	107 ± 19	109 ± 20	129 ± 21	116 ± 15	110 ± 20	125 ± 16
Age 2 months Candesartan	130 ± 18*	99 ± 15	125 ± 13	132 ± 8	130 ± 14	111 ± 15	111 ± 19
Age 21 months Vehicle	108 ± 22*	87 ± 26	109 ± 26	108 ± 18	111 ± 24	102 ± 23	101 ± 16
Age 21 months Candesartan	96 ± 15*	91 ± 16	118 ± 20	111 ± 27	98 ± 17	95 ± 5	109 ± 10

Systolic blood pressure [mmHg] during five posttraumatic days was within physiologic range and not affected by candesartan treatment. At time point 0 that represents intraoperative measurement immediately after CCI induction *under general anesthesia* (in italic characters) systolic blood pressure was higher in young than in old mice (*p < 0.05). Following measurements were performed in awake animals.

Perioperative arterial blood gases were determined 15 min after CCI under general anesthesia in 2 and 21-months-old mice (*n* = 6, respectively) with blood gas analyzer ABL800 Basic (Radiometer Medical ApS, Brønshøj, Denmark) in a parallel investigation, which has been published in 2012 by Timaru-Kast et al. (2012a): pH: 7.32 ± 0.04 and 7.31 ± 0.04, p_a_O_2_: 272 ± 21 and 247 ± 21 mmHg, p_a_CO_2_: 48 ± 4 and 48 ± 5 mmHg for 2 months and 21-month-old mice, respectively. The published data shows that in our standardized anesthesia and operation setting, values were stable and within normal physiological limits in both age groups (Timaru-Kast et al., 2012a).

### *At1a*, *At1b* and *At2* mRNA Expressions Were Age Dependent

In young and aged mice, *At1a*, *At1b* and *At2* mRNA expressions were assessed by qPCR in naïve animals and 1 and 3 dpi (days post injury). In both age groups, *At1a* mRNA expression was not different in naïve mice (*n* = 6, each; young: 4.0 ± 0.6 and old: 3.6 ± 0.1 × 10^−5^ mRNA copies/*Ppia*), whereas naïve *At1b* (young: 1.2 ± 0.2 and old: 2.1 ± 0.1* × 10^−6^ mRNA copies/*Ppia*; *p < 0.05) and *At2* (young: 1.4 ± 0.2 and old: 1.9 ± 0.4* × 10^−4^ mRNA copies/*Ppia*; *p < 0.05) mRNA expression levels were higher in old mice. Data were normalized with cyclophilin A (*Ppia*) and are presented as percentage of naïve expression levels (1 dpi: young and old: *n* = 7 each; 3 dpi: young: *n* = 7; old: *n* = 9; seven survived).

**Figure 1:**
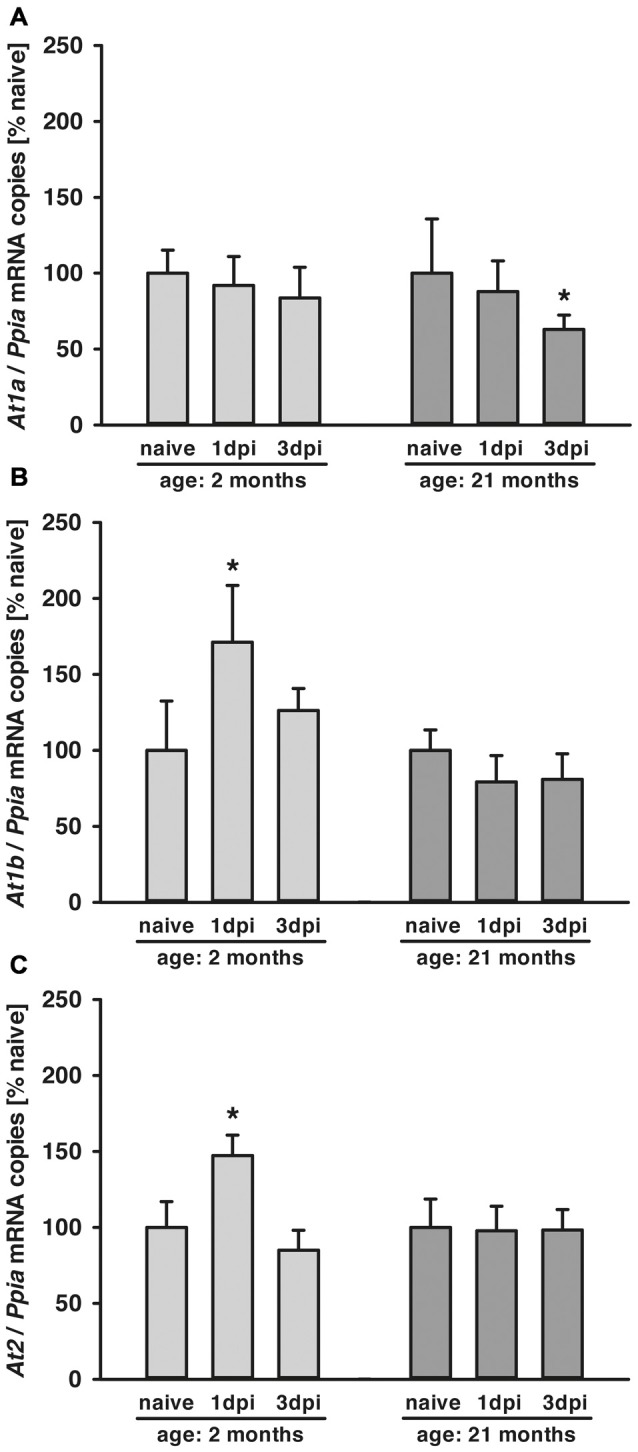
Age-dependent posttraumatic *At1a*, *At1b* and *At2* mRNA expression. **(A–C)** Angiotensin II receptor *At1a*, *At1b* and *At2* mRNA expression was assessed by quantitative real-time PCR (qPCR) in 2 (light gray) and 21-months-old mice (dark gray) in naïve mice and at 1 dpi and 3 dpi (days post injury). Data were normalized with cyclophilin A (*Ppia*) and are presented as percentage of naïve expression levels [*p < 0.05 vs. naive; naïve animals (young and old: *n* = 6 each), 1dpi (young and old: *n* = 7 each) and 3 dpi (young: *n* = 7; old: *n* = 9; seven survived), Mann Whitney Rank Sum Test and Bonferroni-Holm correction; data is presented as mean ± SD].

Posttraumatic cerebral *At1a* expression was not altered in young animals compared to naïve mice during the 3-day observation period. In old animals, expression of *At1a* decreased by 40% at 3 dpi (Figure 1A). In young animals, a posttraumatic increase of *At1b* (70%) and *At2* (50%) expression was found 1 dpi, followed by a drop to naïve levels at 3 dpi. In contrast, *At1b* and *At2* brain expression levels were not changed in older animals for the first 3 days after TBI when compared to naïve control animals (Figures 1B,C).

### Candesartan Did Not Influence Blood Pressure After CCI

The specific AT1 antagonist candesartan is used for the treatment of arterial hypertension. We, therefore, determined its influence on arterial blood pressure. In the present study, low dose (0.1 mg/kg) candesartan was applied, which was shown not to alter blood pressure in young animals in a previous dose-finding study (Timaru-Kast et al., 2012b). Blood pressure monitoring during the five posttraumatic days in awake animals showed that physiologic blood pressure levels in all mice of both age groups (day 1–5 after CCI) was not affected by treatment. Therefore, repeated doses of candesartan did not lower the blood pressure (Table 4).

### Posttraumatic Mortality Was Lower by AT1 Inhibition in Aged Mice

All young animals (age: 2 months) survived the observation time. Aged animals were housed in the animal facility until they reached the age of 21 months. Within this time span, there was a cumulative mortality rate of 45% until 21 months (Timaru-Kast et al., 2012a). The experiments were started for the older group when the age of 21 months was reached. In the angiotensin receptor gene expression time course series, 2/16 (12.5%) of old mice died within the first 3 post-traumatic days. In the treatment study, within the 5-days post-traumatic period, 3/10 (30%) vehicle-treated, and only 1/8 candesartan-treated (12.5%) of older mice died before the end of the observation time.

### AT1 Inhibition Improved Neurological Recovery

**Figure 2:**
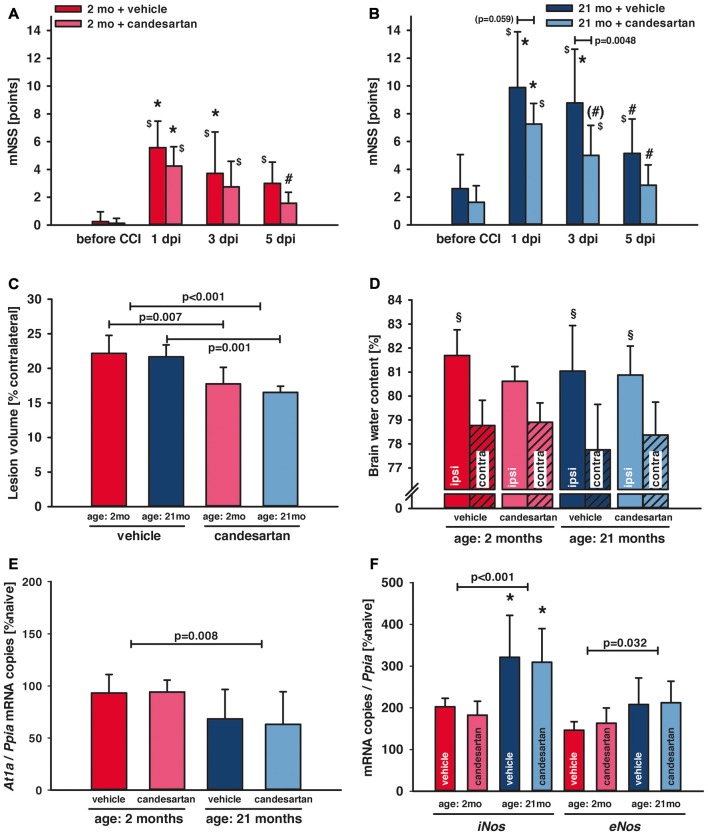
Influence of AT1 inhibition on posttraumatic neurological function, brain damage and brain edema formation. **(A,B)** Neurological assessment was performed before and 1, 3 and 5 days after controlled cortical impact (CCI; 1, 3, 5 dpi) using a modified Neurological Severity Score (mNSS). One day after traumatic brain injury (TBI; 1 dpi) there was a marked neurological impairment in all groups with increased mNSS in old compared to young mice (*p < 0.05 old vs. young) and with lasting elevation of the mNSS in vehicle-treated mice (^$^*p* < 0.05 vs. before CCI). In candesartan-treated young mice there was a neurological recovery with decreasing mNSS at day 5 (^#^*p* < 0.05 vs. 1 dpi). In aged mice, in both treatment groups, mNSS was lower at 5 dpi compared to 1 dpi. However, there was a trend towards faster recovery in candesartan-treated old mice already on day 3 (^(#)^*p* = 0.07). At 3 dpi candesartan treatment reduced neurological impairment compared to vehicle-treatment in old mice (*p* < 0.05). There was a trend in candesartan-treated aged mice to lower impairment already at 1 dpi compared to vehicle treated old mice. After the 5-day observation period, brain lesion volume **(C)** was reduced in candesartan-treated mice in both age groups [*p* < 0.05; young: vehicle and candesartan (*n* = 8 each); old: vehicle (*n* = 10; seven survived) and candesartan (*n* = 8; seven survived)]. **(D)** In a separate set of animals, brain water content was quantified 1 day after the injury. In contrast to vehicle-treated and old mice, in young candesartan-treated mice brain water content ipsilateral to the lesion was not significantly different to contralateral water content (^§^*p* < 0.05 vs. contralateral; young vehicle and candesartan, *n* = 8; old vehicle and candesartan, *n* = 7). Five days after TBI expression of *At1a* **(E)**, *iNos* and *eNos* **(F)** were determined by qPCR. At1a levels were significantly lower in aged animals. iNOS and eNOS showed increased levels in old compared to young mice [*p < 0.05 old vs. young; candesartan: young (*n* = 8); old (*n* = 7) or vehicle young (*n* = 8); old (*n* = 7)]. *At1a*, *iNos* and *eNos* expressions were not altered by treatment. Young animals (2 months, 2mo) are presented in red (dark red: vehicle, light red: candesartan) and old animals (21 months, 21mo) in blue (dark blue: vehicle, light blue: candesartan). Two-way ANOVA with Holm-Sidak’s multiple comparisons test (data is presented as mean ± SD; *p* < 0.05).

Neurological assessment was performed 1 day before and 1, 3 and 5 days after CCI using a mNSS. There was a trend (*p* = 0.06) towards elevated neuroscore before CCI in old [old vehicle: mNSS = 3 ± 2 points, *n* = 10 (seven survived); old candesartan: mNSS = 2 ± 1 points, *n* = 8 (seven survived)] compared to young animals (young vehicle: mNSS = 0 ± 1 points and young candesartan: mNSS = 0 ± 0 points; *n* = 8, respectively). One day after TBI (1 dpi) there was a marked and sustained neurological impairment in all groups with increased mNSS in older compared to young mice (*p* < 0.05) and with longer lasting elevation of mNSS (compared to values before CCI) in vehicle-treated (up to day 5) compared to candesartan-treated mice (until day 3). Independent of age, neurological impairment improved during the following days. Young animals treated with candesartan exhibited reduced functional impairment at 5 dpi (*p* < 0.05; Figure 2A). In aged mice, in both treatment groups, mNSS was lower at 5 dpi compared to 1 dpi. However, when compared to 1 dpi, a trend towards faster recovery was recorded in candesartan-treated older mice already on day 3. Furthermore, compared to vehicle-treated aged mice, AT1 inhibition led to reduced impairment on day 3 in aged candesartan-treated mice (*p* < 0.05) and there was already a trend (*p* = 0.07) for faster recovery with candesartan treatment in old animals as observed already on the 1st day after injury (Figure 2B).

The peri-traumatic BW was assessed as a surrogate parameter of well-being and intake of food and water. Before and until the 5th day after brain trauma, the BW was different between age groups (*p* < 0.001 old vs. young), but not affected by treatment in young mice. However, values were lower during the 5 posttraumatic days in older vehicle-treated mice whereas in old candesartan-treated mice BW remained stable. Therefore, in aged mice, candesartan led to a stabilization of BW after TBI, whereas in vehicle-treated old mice BW decreased over time (*p* < 0.05; Table 3).

### AT1 Inhibition Reduced Brain Damage 5 Days After TBI

Lesion volume was assessed 5 days after CCI in Nissl stained sections. Candesartan treatment reduced lesion volume (−20%) in both age groups (young: vehicle and candesartan *n* = 8, each; old: vehicle *n* = 7/10 survived, candesartan *n* = 7/8 survived; young: 22.2 ± 2.6 and 17.8 ± 2.4*, old: 21.7 ± 1.7 and 16.5 ± 0.9* % contralateral volume; vehicle and candesartan-treated mice, respectively, *p < 0.05; Figure 2C).

### AT1 Inhibition Did Not Influence Brain Edema Formation 1 Day After TBI

Brain water contents were assessed 24 h after CCI (young vehicle and candesartan, *n* = 8; old vehicle and candesartan, *n* = 7). Contralateral (not contused) hemispheres showed similar brain water content compared to naïve. Compared to contralateral values, ipsilateral brain water content was significantly higher in the contused hemispheres of vehicle-treated mice and candesartan-treated older mice (*p* < 0.05). In candesartan-treated young mice ipsilateral increase in brain water content did not reach a level of significance. Although there was a trend towards lower brain edema in candesartan-treated young mice (*p* = 0.07), AT1 inhibition failed to significantly reduce brain edema formation (Figure 2D).

### AT1 Inhibition Did Not Alter *At1a* Expression 5 Days After TBI

To determine the influence of candesartan treatment and age on *At1a* expression levels, tissue samples from naïve mice and injured mice at 5 dpi were investigated (young: *n* = 8 in vehicle and candesartan treatment groups, respectively; old: *n* = 7 in both treatment groups). *At1a* expression remained at naïve levels in young mice. Overall *At1a* expression was lower in aged animals. However, candesartan did not alter *At1a* expression compared to vehicle groups (Figure 2E).

### AT1 Inhibition Did Not Affect Gene Regulation of *iNos* and *eNos* 5 Days After TBI

In order to determine the effect of AT1 inhibition on inducible nitric oxide synthase (iNOS) and endothelial NOS (eNOS), brain tissue samples were examined at 5 dpi. Naïve gene expression of *iNos* (103 ± 18 and 83 ± 25 × 10^−6^ mRNA copies/*Ppia* and *n* = 6 in young and old mice, respectively) and *eNos* (277 ± 120 and 283 ± 47 × 10^−6^ mRNA copies/*Ppia* and *n* = 6 in young and old mice, respectively) were not different. *iNos* mRNA expression increased after injury and was significantly higher in older compared to young mice, indicating a pro-inflammatory state heightened with aging. In vehicle-treated mice, *eNos* expression was also higher in old compared to young mice. AT1 inhibition failed to significantly influence both *iNos* and *eNos* expressions (young: *n* = 8, old *n* = 7 in both treatment groups, respectively; Figure 2F).

#### AT1 Inhibition Reduced Cerebral Inflammation in Young and Old Mice

To investigate the influence of candesartan on cerebral inflammation, we quantified the mRNA expression of inflammatory cytokines and determined the number of activated microglia, T-lymphocytes and neutrophils in the perilesional brain tissue.

Naïve interleukin 1 beta (*Il1b)* mRNA expression was not different between age groups (0.04 ± 0.01 and 0.04 ± 0.03 mRNA copies/*Ppia* for young and old mice, respectively). Naïve expression (*n* = 6 in both age groups) of interleukin 6 (*Il6)* and tumor necrosis factor alpha (*Tnfa)* were higher in aged than in young animals (*Il6*: 0.7 ± 0.2 and 1.1 ± 0.2*; *Tnfa*: 2.0 ± 0.7 and 6.4 ± 0.2* × 10^−5^ mRNA copies/*Ppia* for young and old mice, respectively; *p < 0.05 vs. young), again suggesting a heightened pro-inflammatory state of naïve aged animals.

**Figure 3:**
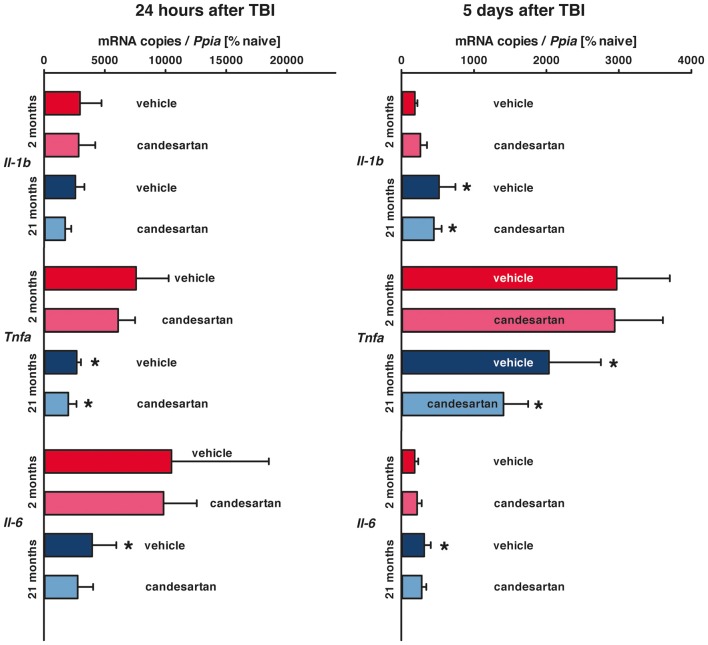
Influence of AT1 inhibition on posttraumatic expression of inflammatory marker genes. *Il1b*, *Il6* and *Tnfa* mRNA expression levels were quantified in the right hemisphere by qPCR in young and old naïve animals (*n* = 6 each), 24 h after insult [candesartan: young (*n* = 8); old (*n* = 7) or vehicle young (*n* = 8); old (*n* = 7)], and 5 days after injury [young: vehicle and candesartan (*n* = 8 each); old: vehicle (*n* = 10; seven survived) and candesartan (*n* = 8; seven survived)]. At both time points after TBI, *Tnfa* expression is increased in young compared to old mice. Whereas expressions of *Il1b* and *Il6* are higher in old mice 5 days after CCI. AT1 inhibition did not affect the expression of these cytokines at both time points after TBI. For better illustration of data different colors were used for each group (2 months: dark red = vehicle, light red = candesartan) and old mice (21 months: dark blue = vehicle, light blue = candesartan treatment); *p < 0.05 21 months vs. 2 months; two-way analysis of variance (ANOVA) with Holm-Sidak’s multiple comparisons test; data is presented as mean ± SD.

After trauma (1 dpi: young *n* = 8 in both treatment groups, old: *n* = 7 in both treatment groups; 5 dpi: young *n* = 8 in both treatment groups, old: vehicle *n* = 10, seven survived, candesartan *n* = 8, seven survived), *Il1b*, *Il6* and *Tnfa* expression increased both in young and old mice with peak expressions at 1 dpi (Figure 3). However, at 1 dpi expression levels of *Tnfa* and *Il6* were higher in young compared to aged animals. Whereas at 5 dpi, *Il1b* and *Il6* expressions were higher in old compared to young mice. Although there was a trend to lower cytokine expression levels with AT1 blockage in old mice, candesartan treatment did not significantly influence the mRNA expression of these cytokines at either time point in both age groups.

**Figure 4:**
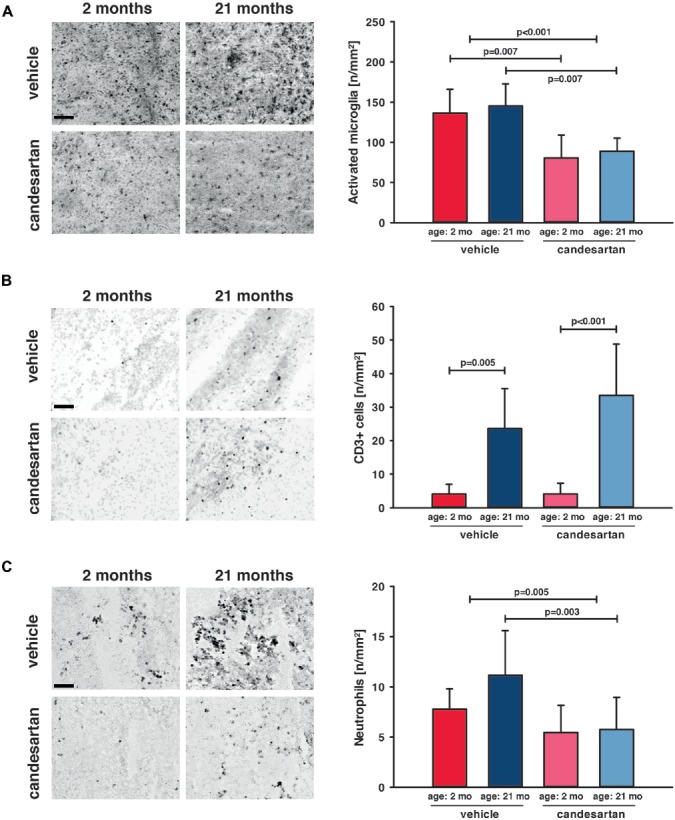
AT1 inhibition reduced cerebral inflammation in young and old mice. Microglial activation and leucocyte infiltration were analyzed 5 days after brain injury. In the left column are representative immunohistochemistry images of stained sections of each age and treatment group (scale bar: 100 μm): Iba-1 **(A)** as a marker for activated microglia, CD3^+^ (**B**) as a marker for T-lymphocytes and Gr1 **(C)** as a marker for neutrophil granulocytes. In the right column, the results are presented as the number of cells/mm^2^ (n/mm^2^). Candesartan treatment reduced microglial activation in both age groups and did not affect T-lymphocyte infiltration. Aged mice showed a higher level of T-lymphocyte infiltration than young mice, independent of treatment. Invasion of neutrophil granulocytes was reduced in both age groups taken together and in old mice by candesartan treatment. However, the reduction of neutrophil granulocyte infiltration did not reach a level of significance in young candesartan-treated mice. For better illustration of data different colors were used for each group (2 months: dark red = vehicle, light red = candesartan) and old mice (21 months: dark blue = vehicle, light blue = candesartan treatment); young: vehicle and candesartan (*n* = 8 each); old: vehicle (*n* = 10; seven survived) and candesartan (*n* = 8; seven survived); two-way ANOVA with Holm-Sidak’s multiple comparisons test; data is presented as mean ± SD.

To determine the impact of candesartan on the cellular level, we quantified the number of perilesional Iba-1 positive cells as a marker for microglia activation 5 days after CCI [young: vehicle and candesartan (*n* = 8 each); old: vehicle (*n* = 10; seven survived) and candesartan (*n* = 8; seven survived)]. Independent of age AT1 inhibition significantly reduced the number of Iba-1-positive cells by −40% (Figure 4A). Next, we investigated if T-lymphocytes and neutrophil infiltration into the brain lesion are influenced by candesartan using antibodies specific for CD3 or Ly-6G (Gr1), respectively. T-lymphocyte infiltration was 6–8-fold higher in aged compared to young animals (*p* < 0.05). However, in both age groups, candesartan did not influence T-lymphocyte infiltration (Figure 4B). By contrast, the number of brain-infiltrating neutrophil granulocytes was significantly reduced by candesartan in older mice and invasion of neutrophil granulocytes was reduced by AT1 inhibition when the factor age is disregarded (in both age groups taken together). However, in young candesartan-treated mice, the reduction of neutrophil granulocyte infiltration did not reach a level of significance (Figure 4C).

**Figure 5:**
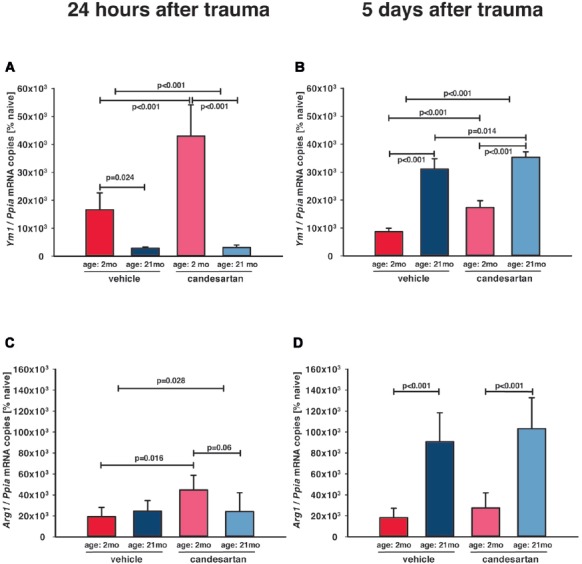
Influence of AT1 inhibition on posttraumatic expression of M2a microglia markers. Anti-inflammatory M2a polarization of microglia was assessed with the M2a markers arginase (*Arg1*) and chitinase-like protein 3 (*Ym1*) in the ipsilateral hemisphere by qPCR in young and old naïve animals (*n* = 6 each), 24 h after insult [**A, C**; candesartan: young (*n* = 8); old (*n* = 7) or vehicle young (*n* = 8); old (*n* = 7)], and 5 days after injury [**B, D**; young: vehicle and candesartan (*n* = 8 each); old: vehicle (*n* = 10; seven survived) and candesartan (*n* = 8; seven survived)]. In young mice, there was an early increase of *Ym1* expression. AT1 inhibition with candesartan showed an enhanced up-regulation of *Ym1* at both time points and of *Arg1* at 1 day after TBI in young animals. In aged mice, both markers were up-regulated later at 5 days after TBI, and this up-regulation of *Ym1* was increased by candesartan treatment. For better illustration of data different colors were used for each group (2 months: dark red = vehicle, light red = candesartan) and old mice (21 months: dark blue = vehicle, light blue = candesartan treatment); two-way ANOVA with Holm-Sidak’s multiple comparisons test (*p* < 0.05); data are presented as mean ± SD.

Increase of M2-like markers, especially M2a-polarization, are associated with neuro-regeneration and improved cognitive and histopathological outcome (Donat et al., 2017), and therefore, anti-inflammatory M2a microglia polarization was assessed by gene expression analysis of the M2a phenotype markers arginase (*Arg1*) and chitinase-like protein 3 (*Ym1*; Chhor et al., 2017). Naïve (young and old, *n* = 6, respectively) *Arg1* (127 ± 56 and 94 ± 6 × 10^−6^ mRNA copies/*Ppia*) and *Ym1* (62 ± 25 and 60 ± 4 × 10^−6^ mRNA copies/*Ppia*; young and old mice, resp.) expression levels were not different between age groups and very low in naïve animals without injury. At all post-traumatic time-points, M2a marker expressions were higher in the injured brain tissues compared to corresponding tissue samples from naïve animals (Figure 5). In young mice, there was an early marked upregulation of *Ym1* 24 h after TBI (Figure 5A), which decreased by 5 dpi (Figure 5B). In aged animals, *Ym1* mRNA expression levels were significantly lower at 1 dpi compared to young animals (Figure 5A), whereas at 5 dpi expression levels of *Ym1* and *Arg1* were 2-fold higher in old compared to young animals (Figures 5B,D). Candesartan treatment increased the post-traumatic expression levels of *Ym1* in young animals at 1 and 5 dpi, indicating an enhanced change of microglia polarization to M2a (Figures 5A,B). However, AT1 inhibition elevated *Arg1* at 1 dpi only in young mice (Figure 5C). At 5 dpi, the strong elevation of *Arg1* in old compared to young mice was independent of candesartan treatment (Figure 5D). Though, up-regulation of *Ym1* at 5 days after TBI was markedly increased in candesartan-treated mice of both age groups. The data indicate a delayed change of microglia polarization to the M2a type in aged animals, that may be boosted by AT1 inhibition.

## Discussion

The present study shows for the first-time an age-dependent mRNA regulation of the angiotensin II receptors *At1a*, *At1b* and *At2* after TBI. Despite these differences, our results demonstrate that posttraumatic AT1 inhibition improves neurological recovery, reduces histological brain damage and limits immune response in both age groups. This effect can be likely attributed to a diminished microglia activation and neutrophil infiltration. Our data further suggest that AT1 inhibition increases anti-inflammatory microglia M2a polarization in young and in aged animals.

Despite a worse clinical outcome in aged TBI patients, one major oversight in the field of TBI research is the paucity of preclinical studies in aged animals, outside of the 3–6 months range (Xiong et al., 2013). The median life expectancy of C57Bl6 mice is 18–24 months. We chose 21-months-old mice in order to investigate the impact of age in animals which can be regarded as geriatric. In a recent study, we demonstrated that the brains of old naïve mice are about 8% smaller compared to young, suggesting age-related brain atrophy (Timaru-Kast et al., 2012a). Aged animals showed a high mortality rate and worse neurological function, but absolute and relative (percentage of contralateral) lesion volumes were not different between ages (Timaru-Kast et al., 2012a). Therefore, in the present study, absolute and relative brain injury volumes were analyzed to rule out an effect by brain atrophy and demonstrated no difference between young and old vehicle-treated mice.

AII mediates the well-known physiological effects of vasoconstriction and blood pressure regulation in cardiovascular disease mainly by AT1. AT1 mediates neuroinflammation and vasoconstriction, aggravating secondary brain damage. AT1 activation is also involved in different pathophysiological neuronal alterations, including neurodegenerative diseases, neuronal injury and cognitive disorders (Culman et al., 2002; Saavedra et al., 2004; Timaru-Kast et al., 2012b; Villapol et al., 2012; Ongali et al., 2014; Villar-Cheda et al., 2014; Hamel et al., 2016; Trigiani et al., 2018). AT1 mediated signaling enhances neuroinflammation and is relevant in the pathogenesis of age-associated degenerative diseases (Rey et al., 2007). In several recent Alzheimer’s Disease studies in mice, AT1 inhibition reestablished normal brain perfusion and vasodilatory function mediated by endothelial and smooth muscle cells and nitric oxide. However, there is a growing body of evidence that restoring cerebrovascular function in chronic neuro-inflammation mouse models is not the only protective mechanism, as AT1 inhibition can also restore cerebrovascular and cognitive function through pleiotropic effects (Hamel et al., 2016). Specifically, recent findings suggest that AT1 inhibitors act directly on neuronal pathways involved in memory formation, hence, working simultaneously albeit independently on neuronal and vascular targets (Hamel et al., 2016). The AT1 inhibitor losartan exerted potent preventive and restorative effects on Alzheimer’s Disease hallmarks, possibly by mitigating AT1-initiated oxidative stress (Ongali et al., 2014). In a recent study, with a murine Alzheimer’s Disease model, candesartan exerted pleiotropic beneficial effects with sustained reduction of cerebrovascular and cognitive deficits after short and long-term treatment. Candesartan largely restored endothelial and smooth muscle function and reduced neuroinflammation. However, in this study, effects were limited by blood pressure effects with the applied dosage of candesartan (Trigiani et al., 2018). Based on our experience, in young mice, repeated treatment with low dose candesartan after TBI did not affect blood pressure. In the present study, neither young nor old mice showed lower blood pressure by repeated low-dose candesartan treatment.

Similar to other neurodegenerative diseases, neuroinflammation with immune cell infiltration and persistent activation of microglia may contribute to poor functional and cognitive recovery in the aged mice following TBI (Popa-Wagner et al., 2011; Timaru-Kast et al., 2012a; Cherry et al., 2014; Hardeland et al., 2015). In the present study, the number of activated perilesional microglia was independent of age. In the acute phase after TBI, two phenotypes of activated microglia could be distinguished (Colton, 2009; Kumar et al., 2016). The classic activation pathway (M1) is characterized by the production of inflammatory cytokines (e.g., IL-1β, IL-6 or TNFα) and reactive oxygen species (ROS) and is associated in aged mice with increased lesion size and neurodegeneration (Kumar et al., 2013). In the alternative activation state (M2), microglia take on an anti-inflammatory phenotype (Gordon and Martinez, 2010). The microglia M2 phenotype can be subclassified into M2a (regenerative), M2b (mixed state) and M2c (immunomodulatory; Xu et al., 2017), with different mediators, receptor profiles and phenotypic markers, which share the capability to downregulate inflammation and activate tissue repair and debris clearance (Mantovani et al., 2004; Varin and Gordon, 2009). The first alternative activation state of the anti-inflammatory microglia subtype is the M2a subtype and is triggered by IL-4 and IL-13 (Bell-Temin et al., 2015). Several studies demonstrate a mixed expression pattern of different markers associated with both M1 and M2 phenotype in the acute phase after TBI (Kumar et al., 2016). The M2a subtype is associated with neuro-regeneration and improved cognitive and histopathological outcome (Bell-Temin et al., 2015; Donat et al., 2017). Therefore, in the present study, the gene expression for the M2a markers ARG1 and YM1 were investigated (Chhor et al., 2017). ARG1 converts L-arginine into prolines and polyamines, which is required for tissue remodeling and wound healing (Munder, 2009; Cherry et al., 2014). ARG1 and iNOS are competing directly for L-arginine. Thus, elevated ARG1 may also be a protective regulation targeting elevated iNOS in aged brains. YM1 is a heparin-binding lectin, which prevents degradation of extra cellular matrix components (Chang et al., 2001; Cherry et al., 2014). Twenty-four hours after insult, *Ym1* expression was higher in young compared to aged mice, whereas 5 days after trauma, *Arg1* and *Ym1* expressions were higher in old compared to young mice. The present study, reveals for the first time that in aged mice, expressions of regenerative M2a markers *Ym1* and *Arg1* (Morganti et al., 2015, 2016) are increased during the first 5 days after TBI, whereas in young mice, in accordance with recent data, the expression of M2-like markers is reduced in the subsequent subacute phase after TBI (Donat et al., 2017). Taking into consideration that posttraumatic expression of proinflammatory cytokines demonstrate an early and prolonged increase in aged animals (Timaru-Kast et al., 2012a), the switch of *Arg1* and *Ym1* expression could indicate a delayed pro-regenerative response in the aged animals.

The present study shows higher perilesional T-cell count in aged mice compared to young animals 5 days after TBI, indicating an age-dependent immune response pattern. However, in a previous study, we demonstrated a marked perilesional increase of T-cells infiltration in young animals compared to old mice, 3 days after TBI (Timaru-Kast et al., 2012a). The comparison of the present results (5 days after TBI) with our published data set (3 days after TBI) reveals a differential regulation. It shows that the number of perilesional T-cells decreased in young mice from 165 to 4/mm^2^ from 3 days (non-treated) to 5 days (vehicle treated) after CCI, whereas in aged mice the number decreased from day 3 to day 5 after TBI from 41 to 24/mm^2^ (Timaru-Kast et al., 2012a).

In humans, two different types of AT (AT1 and AT2) are expressed in the brain. However, in the murine genome *At1* is encoded twice: *At1a* and *At1b*. The two subtypes have 95% identical amino acid sequences and are similar in ligand binding and activation properties, but they differ in distribution and transcriptional regulation (Lenkei et al., 1997). AT1A predominates in the cortex, AT1B in the pituitary gland and in immune cells (Lenkei et al., 1997; Stegbauer et al., 2009). The present study reveals age-dependent differences in posttraumatic expression of *At1a*, *At1b*, and *At2*. While in old animals *At1a* is downregulated 3 and 5 days after TBI, *At1b* and *At2* are upregulated in young mice within 24 h after trauma and return to naïve levels after 3 days. We hypothesize that this differential regulation could be part of an age-dependent rescue mechanism to limit the harmful effects of AT1A activation (Li et al., 2005; Timaru-Kast et al., 2012b). These results are consistent with recent findings demonstrating that aged rats are not able to increase the expression of AT2 in response to dopamine depletion and dopamine receptor downregulation (Villar-Cheda et al., 2014). In line with this data, candesartan did not affect RAS gene regulation after CCI in either age groups (Timaru-Kast et al., 2012b).

AII is one of the most important triggers for inflammation and oxidative stress *via* AT1 and plays a key role in inflammation (Villar-Cheda et al., 2014). AT1 is widely expressed in the mature central nervous system, mainly in neurons, vascular endothelial, smooth muscle cells and astrocytes (Saavedra, 2005). Activation initiates the production of chemokines, cytokines, and adhesion molecules, which contribute to the migration of inflammatory cells into the lesion (Suzuki et al., 2003). Expression of AT1 on residential microglia is induced by AT1 signaling that leads to subsequent activation of microglia, enhanced inflammatory response and finally to the progression of neurodegeneration (Villar-Cheda et al., 2014). AT1 is also expressed on immigrating immune cells, like neutrophil granulocytes, macrophages and T-cells (Ito et al., 2001; Guzik et al., 2007).

In the present study AT1 blockade mediated brain damage reduction was accompanied by a decrease of microglial activation in young and aged mice and by a reduction of neutrophil granulocyte infiltration predominantly in aged mice. The number of activated microglia was 1.6–1.7 times higher in the vehicle than in candesartan-treated animals in both age groups. Therefore, the present study confirms recent findings in candesartan-treated young adult mice, showing a reduced number of neutrophil granulocytes and activated microglia at 3 days after TBI (Villapol et al., 2015).

Several studies demonstrated that the phenotype of microglia is important for their influence on neuroinflammation (Febinger et al., 2015). The microglia RhoA/Rho kinase pathway is activated *via* AT1 (Villar-Cheda et al., 2012; Labandeira-Garcia et al., 2013; Rodriguez-Perez et al., 2013) and TNFα was shown to induce neuronal loss *via* microglia activation and phagocytosis of neurons (Neniskyte et al., 2014). In the present study, microglia polarization was affected by AT1 inhibition in young and aged mice, demonstrating an enhanced switch to protective anti-inflammatory M2a phenotype. In contrast to young animals, the candesartan dependent M2a switch was only present at 5 days after TBI in aged animals. A recent study showed that expression of CD62L on human neutrophils is modulated by AT1 receptors, reducing the immigration of neutrophils by pathways involving extracellular signal-regulated kinases 1 and 2 (ERK1/2), mitogen-activated protein kinase (MAPK), phosphatidylinositol 3-kinase, and calcineurin (CaN; Vega et al., 2010). Therefore, AT1 inhibition may have a direct anti-inflammatory action on invading neutrophils and resident activated microglia (Villapol et al., 2015). Perilesional T-cell migration was not affected by AT1 blockade. Neuroprotective mechanisms of AT1 inhibition is therefore independent of adaptive lymphocyte reaction. In summary, the putative mechanism of AT1 inhibition mediated neuroprotection could be a reduction of cerebral inflammatory response of the innate immune system with reduced resident microglia activation as well as decreased invasion of neutrophil granulocytes.

Signaling of AT1 in immune cells induces inflammatory responses by NADPH oxidase activation and NF-κB dependent transcription of several pro-inflammatory cytokines (Borrajo et al., 2014b; Rodriguez-Perez et al., 2015). This leads to the stimulation of several kinases that participate in the propagation of inflammatory responses and apoptotic pathways (Villapol et al., 2015). It has been shown in microglia that AT1 mediated release of TNFα and NF-κB translocation can be reduced by AT1 antagonists (Borrajo et al., 2014a,b). AT1 blockade reduced microglia response, oxidative stress and dopaminergic degeneration in aged rodents (Labandeira-Garcia et al., 2011; Villar-Cheda et al., 2014). In a recent study, we observed a lower cytokine expression by AT1 inhibition 12 h after TBI (Timaru-Kast et al., 2012b). However, in the present study, there was only a trend to lower *Il1b* and *Il6* levels in aged mice at 24 h after TBI. Cytokine expression 5 days after TBI was not influenced by AT1 blockade. A recent study showed that an early acute increase in TNFα levels is followed by a decrease in the next days to control values (Borrajo et al., 2014a), whereas we recently showed an early and long-lasting cerebral inflammation up to 3 days after insult (Timaru-Kast et al., 2012a). Therefore, the lack of effect of AT1 inhibition on inflammatory marker gene expression in the present study could also be due to lower cytokine mRNA levels at 5 days after CCI or the limited impact of candesartan treatment on inflammatory cytokine mRNA expression. In the present study, the expressions of the nitric oxide synthases iNOS and eNOS were assessed. Inducible NOS is elevated in the inflammatory state, as compared to the constitutively expressed eNOS and neuronal NOS (nNOS). However, *iNos* and *eNos* were up-regulated after TBI and regulation were not influenced by AT1 inhibition.

The neurological outcome was determined by a mNSS (modified after Tsenter et al., 2008) 1 day before and after TBI (24, 72, and 120 h after CCI). The mNSS evaluates mainly motor function with a focus on general behavior, alertness, motor ability and balance and does not measure cognitive dysfunctions (Tsenter et al., 2008; Thal et al., 2013). There was a trend showing elevated neuroscore levels before CCI in old compared to young animals that were likely due to higher BW and loss of alertness, balance and motor ability in old compared to young mice. As a result of the brain trauma, there was a marked and sustained neurological impairment in all groups. The impairment was markedly worse in aged mice than in young mice. Independent of age, neurological impairment improved during the following days. Elevation of mNSS was longer lasting and higher in vehicle-treated compared to candesartan-treated mice and functional recovery was improved by candesartan treatment in both age groups. Furthermore, besides earlier improvement of neurological outcome in old mice, AT1 inhibition limited BW loss after TBI in aged mice, as a surrogate parameter of well-being, better food and water intake of these animals.

In order to correct methodological errors during mRNA quantification, differences in RNA quality and quantity and efficacy of the reverse transcription, we used the most frequently applied technique to normalize target gene expression with a cellular maintenance gene, a so-called reference gene that is assumed or proven to be stably expressed in an experimental setting (Bustin, 2002; Huggett et al., 2005). Cyclophilin A was chosen as a single normalizer for both age groups based on our recent work comparing different reference genes in young, 2-months-old and 21-months-old mice after experimental TBI. In young and old mice, cyclophilin A (*Ppia*) was the most robust gene after TBI, thus being most suitable to serve as a single normalizer (Timaru-Kast et al., 2015).

There is a growing body of evidence that the choice of commercial cDNA synthesis kit has a major impact on qPCR results and on comparability between studies. Previous investigations have shown that normalization with a reference gene fails to reduce kit-dependent variations (Garcia-Bardon and Thal, 2016). The solution provided by this study to overcome this limitation is normalization with data from naïve samples. In order to improve the comparability of qPCR data between different samples from different ages and treatment groups and to eliminate qPCR kit dependent differences and limitations, mRNA expression was normalized with the expression of reference gene *Ppia* and presented as a percentage (%) of data from corresponding naïve tissue samples. This procedure should improve the comparability of our mRNA expression data (Garcia-Bardon and Thal, 2016).

In summary, the present study revealed that AT1 inhibition by low dose candesartan treatment improved neurological outcome and reduced brain lesions after TBI in young and old mice. In young mice, the microglia activation was reduced, and the microglial polarization was markedly shifted to the protective M2a phenotype by candesartan, whereas in old mice microglia activation and neutrophil infiltration were more strongly reduced compared with young mice.

## Conclusion

The inflammatory response is believed to play a key role with detrimental effects on posttraumatic brain damage. The present study shows differential changes of *At1a*, *At1b* and *At2* in young and aged healthy mice and after trauma. AT1 inhibition was highly effective in young and old animals to reduce brain damage and improve neurological impairment by reducing microglial response and neutrophil granulocyte infiltration. Thus, we can conclude that AT1 inhibition is an effective neuroprotective strategy against age-related exacerbated cerebral inflammation after TBI. AT1 inhibition is, therefore, a promising therapeutic strategy to limit secondary brain damage after TBI, independent of age. The present data may help to understand the TBI pathophysiology in aged individuals and develop an optimal pharmacologic intervention for the aged population.

## Author Contributions

RT-K and ST contributed to the conception and design of the study, performed the statistical analysis and wrote the first draft of the manuscript. PG, CL, CH, RH and MS wrote sections of the manuscript. All authors contributed to manuscript revision, read and approved the submitted version.

## Conflict of Interest Statement

The authors declare that the research was conducted in the absence of any commercial or financial relationships that could be construed as a potential conflict of interest.

## Footnotes

^1^https://www.pubmed.de/tools/zufallsgenerator

^2^www.mbl.org
